# A systematic review and narrative synthesis of prevalence rates, risk and protective factors for suicidal behavior in international students

**DOI:** 10.3389/fpsyt.2024.1358041

**Published:** 2024-03-14

**Authors:** Maria Veresova, Michelle Lamblin, Jo Robinson, Samuel McKay

**Affiliations:** ^1^ Orygen, Parkville, VIC, Australia; ^2^ Center for Youth Mental Health, The University of Melbourne, Parkville, VIC, Australia

**Keywords:** international students, suicide, suicide prevention, self-harm, risk factors, protective factors, systematic review

## Abstract

International students are a crucial group for suicide prevention efforts. However, no comprehensive review of the prevalence, risk or protective factors for international student suicide has been conducted, complicating prevention efforts. The current systematic review addresses this issue by identifying the prevalence, risk, and protective factors for suicidal thoughts and behavior in international students enrolled in tertiary institutions. We searched CINAHL, EMBASE, ERIC, Medline, and PsycInfo from inception to November 2023, and 24 papers met the study inclusion criteria. Nineteen studies reported on the prevalence or intensity of suicidal ideation, six reported on the prevalence of suicide attempts, and seven on the prevalence of self-harm. No studies provided data on completed suicides. Studies indicated that international students experienced similar or lower levels of suicidal ideation and self-harm, but more frequent suicide attempts than domestic students. A narrative synthesis of risk and protective factors yielded mixed results, although social isolation, comorbid mental health conditions, and experiences of racism or discrimination were commonly linked to suicidal thoughts and behaviors. The mixed evidence quality, numerous measurement issues, and low number of studies in the literature point to a need for further research on suicide among international students.

**Systematic review registration:**

https://www.crd.york.ac.uk/PROSPERO/, identifier CRD42022307252.

## Introduction

Suicide has been one of the leading causes of death worldwide in the recent years (1–3). Suicidal thoughts and behaviors are particularly prevalent among tertiary education students (4). A significant portion of students in higher education are international students who transition from their primary country of residence to pursue education abroad (5, 6). International students face a unique combination of challenges such as language barriers, cultural adjustment, financial stress, and academic pressure (7). These factors can negatively impact mental health (8, 9) and potentially increase the risk of suicidal thoughts and behaviors. Complicating matters, international students are less likely to seek help for mental health-related struggles compared to domestic students (10). Furthermore, recent investigations into international student suicides highlight that these students’ engagement with mental health services prior to their death is minimal (11).

Despite evidence suggesting that international students may experience multiple risk factors for suicide, there has been little focus on this population when it comes to suicide prevention research (12). A recent scoping review found that there is a lack of high-quality research on this topic, and that no suicide-specific interventions for international students currently exist (13). Encouragingly, broader suicide prevention activities delivered in tertiary education institutions have been shown to improve students’ knowledge and attitudes towards suicide and rates of engagement with mental health services (14). However, such programs may not be appropriate or adapted to international student needs. People from culturally and linguistically diverse backgrounds have distinct perspectives on mental health and suicide, and effective suicide prevention for these groups necessitates tailored strategies (13, 15). Foundational to this is epidemiological research that identifies potential points for intervention. Currently, there is a gap in knowledge regarding the prevalence rates of, and risk factors for, suicidal thoughts and behaviors in international students. This limits our capacity to develop effective, evidence-based suicide prevention interventions for this population.

The present review aims to address these gaps by synthesizing the available literature on suicide and suicide related behaviors in international students by answering the following questions: a) What is the prevalence of suicidal ideation, suicide attempts and self-harm in international students, and b) What are the risk and protective factors that influence suicides, suicidal ideation, attempts, and self-harm in international students?

## Materials and methods

The protocol for this review was registered on PROSPERO (CRD42022307252). The summary of prevalence rates of suicidal thoughts and behaviors was reported in accordance with the Preferred Reporting Items for Systematic Reviews and Meta-Analyses (PRISMA) guidelines (16). Because studies that examined risk and protective factors for suicidal thoughts and behaviors varied considerably in their methodologies and variables of interest, narrative synthesis was deemed most appropriate to summarize the findings. The narrative synthesis approach was guided by Rodgers et al. (17).

### Eligibility criteria

The inclusion criteria comprised peer-reviewed empirical studies that a) identified rates of suicidal ideation, attempts, or self-harm, or b) examined risk or protective factors for suicide, suicidal ideation, attempts, or self-harm in international students.

International students were defined as tertiary education students of any age enrolled in an educational program in a different country to where they typically reside or hold citizenship. Studies were included if international student participants made up the entire sample or were identified as a subset of a broader sample with their data reported separately. Studies from any publication year and geographical region were included. Case studies and qualitative studies were excluded as the current review focused specifically on quantitative evidence. Sources that were not in English were excluded due to resource limitations.

### Search strategy and data sources

A comprehensive search strategy was developed with support from a librarian to identify published articles relevant to the research questions. Two platforms (EBSCOhost & OVID) covering five databases (CINAHL, EMBASE, ERIC, Medline, & PsycINFO) were searched to cover a variety of disciplines (e.g., health, psychology, and education) relevant to the research topic. Search terms were adapted for each platform and database. Searches included studies from the database inception to 8 November, 2023. Reference lists from identified studies were checked for any additional relevant studies that may have been missed during the electronic search. The full search strategy is reported in **Appendix A**.

### Reference management and screening

All search results were imported into Endnote, duplicates were checked and removed, and the full reference list was generated and uploaded to the systematic review screening tool Covidence (18). Additional duplicate checks were undertaken in Covidence. SM performed the database search, combined the records, and ran the duplicate removal processes. Titles and abstracts were independently screened for relevance to the research question and inclusion criteria by MV and SM. Full texts of included articles were then screened for more detail by the same two reviewers. All full-text studies that were excluded had their reasons for exclusion recorded. Discrepancies in screening decisions between the two reviewers were discussed until consensus was reached.

### Data extraction and analysis

A tailored data extraction template was created (**Appendix B**) to extract incidence of suicide, prevalence rates of suicide attempts and self-harm, and prevalence rates and intensity levels of suicidal ideation. In this review, a risk or protective factor was conceptualized as any variable that was defined, measured, and tested for a statistically significant relationship with suicide, suicidal ideation, attempts, or self-harm, through any type of analysis (e.g., correlation or regression). SM and MV independently performed data extraction for each included article, and subsequently verified the extraction results for accuracy.

### Quality assessment

Quality assessment was performed using the Checklist for Assessing the Quality of Quantitative Studies (CAQQS) (19) as this tool provides a comprehensive evaluation of the methodological quality of research. All studies were included in the final review regardless of how they scored on the CAQQS.

## Results

### Selection of sources of evidence

**Figure 1:**
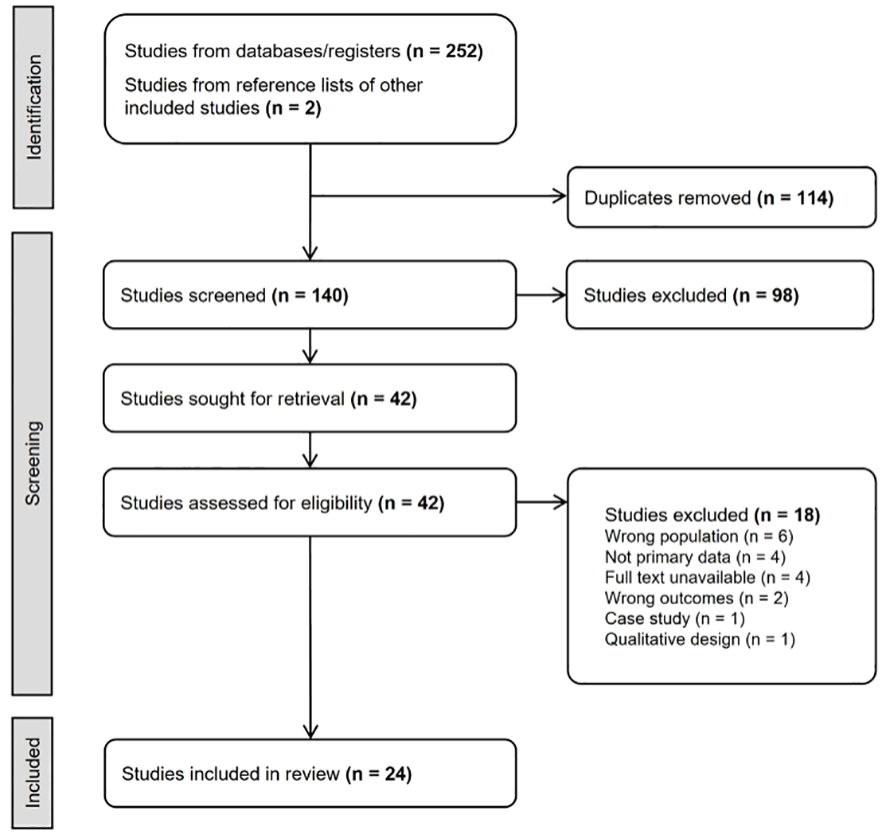
The PRISMA flowchart describing the process of systematic search and study selection.

The process of searching, retrieving, and screening studies is described in **Figure 1**. The inter-rater reliability between the two reviewers was κ = 0.66 [substantial (20)] for the abstract screening and κ = 0.85 (almost perfect agreement) for the full-text screening. Twenty-four studies were included in the final review, and their key characteristics and quality assessment scores are reported in **Table 1**.

**Table 1:** Summary of included studies and relevant key findings.

Study ID, country, & quality appraisal	Study aims	Participants	Country of origin of international students	Outcome measures	Results: rates and levels of suicidal ideation, attempts and self-harm in international students (incl. comparisons with domestic students where available)	Results: risk and protective factors
21 Taiwan CAQQS: 77.3%	To investigate how international and local students in Taiwan differ in terms of COVID-19 susceptibility perception, resource sufficiency, information seeking, support satisfaction, and mental health during the COVID-19 outbreak.	529 university students. International = 216 (Age M = 28.45, SD = 6.41, 54.17% female, 45.4% male). Domestic (Taiwanese) = 313 (Age M = 23.85, SD = 4.91, 37.38% female).	Not reported	Suicidal ideation – 1 item specifically developed for the study: “In the past seven days, did you have a suicidal idea” on a 5-point scale from 1 (Not at all) to 5 (Very severe), range = 1-5.	International student suicidal ideation: M = 1.14, SD = 0.49. NS different to domestic students.	Residing with family, perceived susceptibility to COVID-19, and perceived insufficiency with resources needed to prevent COVID-19 were significant predictors of suicidal ideation among international students.
22 China CAQQS: 86.4%	To assess distress levels between Chinese international and domestic students, exploring how everyday discrimination affects distress and identifying moderating factors.	686 college students. International students = 381 (Age M = 22.29, SD = 2.7, 57.7% female, 42.3% male).	American (61.9%), Australian (10.5%), Canadian (6.3%), British (5.2%), and other countries (16.0%).	Suicidal ideation - PHQ-9 item 9: “Thoughts that you would be better off dead, or of hurting yourself” on a 4-point Likert scale from 0 (Not at all) to 3 (Nearly every day). Measured in the past 2 weeks.	28.6% of international students reported suicidal ideation at least several days (21.8% [Several days]; 4.5% [More than half the days]; 2.4% [Nearly every day]) during the past two weeks.	Not reported
23 The United States CAQQS: 81.8%	To examine the relationship between public stigma towards mental health treatment and suicidality across diverse ethnic groups of college students.	153,635 college students. Asian International students = 7054. 91.56% aged 18-30. 51.56% female. Other student ethnic groups = 146,581. 90.97% aged 18-30. 58.89% female.	Not reported	Suicidal ideation – “Ever seriously considered ending life within the past 12 months?” (Yes/No) Suicide planning – “Ever made a plan to end life within past 12 months?” (Yes/No) Suicide attempt – “Ever attempted to end life within past 12 months?” (Yes/No)	6.34% of Asian international students endorsed past-year suicidal ideation, 2.12% endorsed a past-year suicide plan, and 1.18% reported a suicide attempt in the past year. Asian international students had significantly higher suicide attempt rates but significantly lower suicidal ideation/planning rates compared to white students.	Among Asian international students, the positive association between perceived public stigma and suicidal ideation, as well as suicide attempts, was stronger than in white students.
24 The United States CAQQS: 86.4%	To investigate differences in clinical features such as suicidal thoughts, suicide attempts, and NSSI across various socio-demographic student groups attending psychiatric emergency service.	725 students (Age M = 22, SD = 3.97, 56% female) with 63 (8%) international students (65.08% female, 34.9% male).	Not reported	C-SSRS was used for all suicide-related outcomes. Suicidal ideation – 5 items of the C-SSRS (dichotomized to Yes/No) in the past week. Suicide attempts - Past week attempt history (Yes/No), lifetime multiple attempt history (one or less/two or more). NSSI - Past week NSSI (Yes/No), lifetime multiple NSSI (one or less/two or more).	12.7% reported a past week suicide attempt. NS different to non-international students. 14.3% reported lifetime multiple attempts. Significantly higher than non-international students. 46% reported suicidal ideation. NS different to non-international students. 12.7% and 25.4% reported past-week and lifetime multiple NSSI respectively. NS different to non-international students.	Not reported
25 The United States CAQQS: 81.8%	To explore mental health outcomes in Asian international students based on perfectionistic family backgrounds.	190 Asian international students (Age M = 24.68, SD = 4.92, 54% female, 46% male).	China (52%), India (15%), South Korea (8%), Indonesia (5%), Taiwan (4%).	Suicidal ideation – measured by the SIS, which consists of 10 items on a 5-point Likert scale from 1 (Rarely or none of the time) to 5 (Most or all of the time), range = 10-50.	SIS scores for the sample: M = 11.3, SD = 2.59.	Depression, generalized anxiety, social anxiety, eating concerns, hostility, maladaptive perfectionism, academic stress, and family discrepancy between desired and actual educational performance were positively associated with suicidal ideation in this sample.
26 Canada CAQQS: 81.8%	To compare risk factors and associated mental health and academic outcomes between international and domestic students.	2991 university undergraduate students. International = 297 (10.4% aged 17 or below, 84.5% aged 18-19, and 5.1% aged 20 or above, 64.7% female, 33.7% male). Domestic = 2694. (20.3% aged 17 or below, 73.4% aged 18-19, and 6.3% aged 20 or over, 66.9% female.)	The majority of international students were from Asia, with 63% of participants at baseline and 45% at 6-month follow-up from China.	Lifetime self-harm, suicidal ideation, and suicide attempts were assessed at school entry and follow-up (in the past 6 months) using questions from the C-SSRS. Specific items and scoring not reported.	At baseline, international students reported lifetime suicidal thoughts (males = 20.5%, females = 35.3%), self-harm without intent to die (males = 12.1%, females = 16.8%), and suicide attempts (males = 7.2%, females = 9.6%). At 6-month follow-up, international students reported suicidal thoughts (males = 7.5%, females 15.3%), self-harm without intent to die (males 5.0%, females = 7.1%), and suicide attempts (males = 2.5%, females = 4.7%). International students were more likely to have made a suicide attempt than domestic students.	Not reported
27 The Netherlands CAQQS: 86.4%	To compare mental health outcomes in domestic and international university students during the COVID-19 pandemic in two cohorts (2020 and 2021) with self-reported stress and/or mood problems.	349 university students. 169 international and 180 domestic students. 2020 cohort = 207 (Age M = 20.3, SD = 2.19, 82% female). 2021 cohort = 142 (Age M = 21.1, SD = 5.61, 80% female).	2020 cohort: Europe (90%), Asia (6%), South America (1%), North America (2%). 2021 cohort: Europe (92%), Asia (2%), South America (2%), North America (2%), Africa (1%).	Suicidal ideation – Measured by the BSSI, which contains 21 items capturing past-week suicidal ideation scored on a 3-point scale from 0-2, range = 0-42.	International students had significantly higher past-week suicidal ideation levels (M = 3.11 SD = 5.86) than domestic students (M = 1.23 SD = 3.54).	Not reported
28 The United States CAQQS: 63.6%	To explore gender differences in Chinese international college students’ mental health and substance abuse during the COVID-19 pandemic.	1010 Chinese international students (Age M = 20.19, SD overall = 1.5, 47.03% female, 53.0% male).	China	The following questions were specifically developed for the study: 1. Have you ever thought or did self-injury in the past year? (Behaviors such as pulling hair, cutting wrists/arms, scratching, and fingernail biting, etc.) (Yes/No) 2. Have you ever thought or did suicide behave in the past year? (Yes/No)	23.4% of males and 28.6% of females reported that they had either thought about or engaged in self-harm in the prior 12 months, while 17.9% of males and 20.7% of females reported that they have either thought about or attempted suicide during the past 12 months.	Female international students were more likely to have thought about or engaged in self-harm than males.
29 Australia CAQQS: 90.9%	To examine the extent to which the relationship between stressful life events and suicidal ideation for Asian international students was moderated by levels of loneliness, campus connection, and problem-focused coping.	138 Asian international students (Age M = 21, SD = 1.86, 68% female, 32% male).	Singapore (44.2%), Malaysia (39.9%), Indonesia (8.7%), Hong Kong (3.6%), India (1.4%), Sri Lanka (0.7%) and Thailand (0.7%).	Suicidal ideation - The first 5 items of 19-item of BSSI, rated on a scale 3-point scale from 0-2, range 0-10. Measured in the past week.	Past-week suicidal ideation: M = 0.38, SD = 0.83.	Loneliness, low campus connectedness, and stressful life events were positively associated with suicidal ideation. The relationship between suicide ideation and stressful life events was stronger for individuals with high levels of loneliness. Conversely, the relationship between stressful life events and suicide ideation was weaker for individuals with high levels of campus connectedness and those with greater problem-focused coping skills.
30 The United States CAQQS: 81.8%	To examine experiences of victimization and campus safety among Asian American and Asian international undergraduate students.	2385 undergraduate students. Asian international = 470 (Age M = 20.4, SD = 2.01, 40.2% male, 59.1% female, 0.6% other). Domestic (Asian American) = 1915 (Age M = 19.88, SD = 1.92, 34.1% male, 65.7% female, 0.3% other).	Specific Asian countries not reported.	Suicidality - Respondents were asked if they had seriously considered suicide or attempted suicide. Response options included “No, never”; “No, not in the past 12 months”; “Yes, in the past 2 weeks”; “Yes, in the past 30 days”; and “Yes, in the past 12 months”, but all the categories were collapsed to 0 (no suicidality in past year) and 1 (suicidality in past year).	7.7% of international students reported some form of suicidality (e.g., seriously considered or attempted) in the past year. NS different from domestic student suicidality.	Heterosexual Asian international students were significantly less likely to report feeling suicidal compared to sexual minorities from this group.
31 Georgia CAQQS: 63.6%	To identify the prevalence of anxiety, depression, and suicidality and identify relevant risk and protective factors among university students during the COVID-19 pandemic in Georgia.	984 university students. International = 224 (Age M = 21.5, SD = 2.2, 44.6% male, 54.9% female, 0.4% other). Domestic = 760 (Age M = 20.5, SD = 2.3, 23.7% male, 75.7% female, 0.7% other).	Not reported	Suicidality - Risk Assessment Suicidality Scale. Specific items, subscales, and scoring not reported.	Not reported independently for international and domestic students.	Not reported independently for international students.
32 Japan CAQQS: 72.7%	To examine the effect of sense of connectedness, perceived information inaccessibility and willingness to seek help from informal and formal sources on suicidal ideation.	268 university students (Age M = 20.87). Domestic = 67. 33.96% aged 17-19, 48.13% aged 20-22, 17.91% aged >22. 62.69% female. International = 201. 35.82% aged 17-19, 43.28% aged 20-22, 20.90% aged >22. 63.68% female, 36.3% male.	61.0% of international students were from South-East Asia (Vietnam, Indonesia, Thailand, and Malaysia), 25% were from East Asia (China, Korea, and Taiwan), 9% were from South Asia, and 5% from other regions.	Suicidal ideation - PHQ-9 item 9: “Thoughts that you would be better off dead, or of hurting yourself” on a 4-point Likert scale from 0 (Not at all) to 3 (Nearly every day). Measured in the past 2 weeks.	23.4% of international students reported experiencing suicidal ideation^1^.	Low social connectedness was positively associated with suicidal ideation in international students.
33 The United States CAQQS: 90.9%	To concurrently examine the relationships among discrimination, cross-cultural loss, academic distress, thwarted belongingness, perceived burdensomeness, and suicidal ideation in international students.	595 international students (Age M = 24.57, SD = 4.56, 51.25% male, 47.73% female, 0.5% other).	India (34.8%), China (19%), Vietnam (3.4%), Malaysia (2.4%), Colombia (2.2%), Indonesia (2.0%), South Korea (2.0%), and 67 other countries from every world region.	Suicidal ideation - SIS, which contains 10 items on 5-point Likert scale from 1 (Never or none of the time) to 5 (Always or a great many times), range = 1-5 (normalized). Measured in the past week.	Suicidal ideation scores for the sample: M = 1.30, SD = 0.58, Range = 1-5^2^.	Discrimination, academic distress, cross-cultural loss, and perceived burdensomeness were positively associated with suicidal ideation. Thwarted belongingness correlated with suicidal ideation but did not predict suicidal ideation when placed in a predictive model with other factors.
34 Australia CAQQS: 94.4%	To describe the mental and physical health of a large and representative sample of international students from one university, and the extent to which these students engage in behaviors that are potentially health-compromising.	979 international students. 57% of students were aged 20–24 years. 64% female.	China (23%), Malaysia (23%), Singapore (12%), Indonesia (10%), Hong Kong (8%), The United Kingdom/The United States/Canada (total of 4%), and other European countries (3%).	Self-harm and suicidal ideation and behavior since beginning studies in Australia were examined through Yes/No responses to 3 questions: “Have you deliberately hurt yourself?” “Have you done something because it might harm/kill you?” “Have you thought about taking your own life?”	Deliberately hurt self: 7 (2.0%) males and 28 (4.5%) females. Did something to that could harm/kill self: 3 (0.9%) males and 20 (3.2%) females. Thought about taking own life: 23 (6.7%) males and 70 (11.3%) females.	Students who engaged in self-harm had significantly higher scores on depression, anxiety, stress, and abuse and distress than those who had not self-harmed.
35 Australia CAQQS: 94.4%	To provide a cross-sectional examination of a broad range of health and wellbeing factors affecting the mental health and academic performance of an Australian university student population, using a university-wide anonymous survey.	14,880 university students (Age M = 24.19, SD = 6.88, 63.9% female, 35.6 male, 0.5% other). International = 5,482 (Age M = 23.71, SD = 4.44, 63.6% female, 36.3% male, 0.1% other). Domestic = 9,398 (Age M = 24.46, SD = 7.95, 64.1% female, 35.1% male, 0.8% other).	48.7% (n = 2638) were from China, while others came from various countries/continents including India, Malaysia, Indonesia, Singapore, Hong Kong, Vietnam, Europe, and the United States.	Suicide attempts/self-harm was assessed via one question asking the participant if they have tried to “harm or kill themselves in past 12 months” (Yes/No).	3.9% of international students (173) reported that they have tried to harm or kill themselves in the past 12 months.	Not reported.
36 The United States CAQQS: 77.3%	To examine suicide ideation in international and domestic students using the Interpersonal Theory of Suicide framework.	254 undergraduate students (Age M = 21.1, SD = 1.8, 48.43% female). International = 46. Domestic = 208.	China (n = 21), India (n = 9), Malaysia (n = 4), Kenya (n = 2). Single students indicated the following home countries: Armenia, Bangladesh, Canada, Korea, Mexico, Panama, Russia, Spain, Sweden, and Turkey.	Suicidal Ideation - 7 items specifically developed for use by the National Research Consortium of Counseling Centers in Higher Education. Participants were asked to consider a recent stressful period in the prior 12 months and to indicate whether or not they had any of the following thoughts during that period: (a) This is just all too much (b) I wish this would all end, (c) I have to escape (d) I wish I was dead (e) I want to kill myself, (f) I might kill myself, and (g) I will kill myself. Response options were dichotomized as 1 (true) or 0 (false) and summed with higher scores representing greater suicidal ideation.	Specific levels not reported. NS suicidal ideation levels between international and domestic students.	Low campus belongingness and high family belongingness were associated with high suicidal ideation in international students.
37 The United States CAQQS: 81.8%	To identify risk and protective factors associated with greater emotional distress and suicide ideation among international college students.	334 international students (age range 18-26, 56% female).	Students reported their race/ethnicity as Asian (62%), European (11%), Hispanic/Latino (9.3%), African (4.8%), Middle Eastern (4.8%), and Other (7.8%).	Suicidal ideation - 2 items were modified from the Suicidal Behaviors Questionnaire-Revised (SBQ-R) and the Self-Injurious Thoughts and Behaviors Interview (SITBI). Exact questions and scoring not reported, range = 2-12. Measured in the past year. Suicide attempt - “Have you made a suicide attempt in the past year?” (Yes/No).	Suicide ideation in the past year (M = 2.6, SD = 1.5). Within the previous year, 18.2% of students reported any suicidal thoughts, 5.5% seriously considered suicide, and 4.8% had strong desires to kill themselves. A total of 2% attempted suicide during the past year.	Feelings of entrapment, cultural stress, family conflict, perfectionism, unmet interpersonal needs, and ethnic discrimination were positively correlated with suicidal ideation. Only unmet interpersonal needs remained significantly associated with greater suicidal ideation in a multivariate regression analysis.
38 The United States CAQQS: 81.8%	To examine the relationship between mask-wearing behavior, perceived discrimination, and self-harming thoughts among international students in the US during the COVID-19 pandemic, with a focus on the potential moderation effect of perceived discrimination.	103 international students (Age M = 27.76, age range 19-48, 61.2% female).	Participants were from 38 countries; most were from South Korea (16.5%), China (15.5%), India (10.3%) and Brazil (7.2%).	Self-harming thoughts were assessed by a question adapted and modified from the Youth Risk Behavior Survey: “How often have you had thoughts about harming yourself during the COVID-19 pandemic?” Participants used a 5-point Likert scale ranging from 0 (Never) to 4 (Very often), but the variable was dichotomized to indicate any self-harming thoughts during the pandemic.	18.6% of participants reported self-harming thoughts during the pandemic.	Loneliness and perceived discrimination were positively associated with self-harm thoughts.
39 The United States CAQQS: 95.5%	To investigate the moderating effects of maladaptive perfectionism and perceived discrimination on the relationship between interpersonal risk factors and suicide ideation among Asian international students.	466 Asian international students (Age M = 26.39, SD = 4.99, 49.57% female, 50.4% male).	52.8% Chinese, 14.8% Indian, 8.4% Korean, 6.4% Vietnamese, 4.5% Taiwanese, 2.8% Thai, 2.1% Sri Lankan, 1.5% Indonesian, 1.5% Japanese, 1.3% Malaysian, 1.1% Nepalese, 0.9% Filipino, and 5 other subgroups.	Suicidal ideation – Measured by the SIS, which contains 10 items on 5-point Likert scale from 1 (Never or none of the time) to 5 (Always or a great many times), range = 10-50. Measured in the past week.	Suicidal ideation in the sample: M = 12.99, SD = 5.75.	Depression, discrepancy between desired and actual educational performance (both personal and family), discrimination, and perceived burdensomeness significantly correlated with suicidal ideation. Family discrepancy intensified the relationships of perceived burdensomeness and thwarted belongingness with suicide ideation. Thwarted belongingness correlated with suicidal ideation but did not predict suicidal ideation when placed in a predictive model with other factors.
40 The United States CAQQS: 95.5%	To compare the mental health status of Asian international students in the United States with American students and other international students.	10,731 total participants. Asian international = 3701 (51.2% female). Non-Asian international = 3380 (55.4% female). Domestic = 3649 (51.3% female).	Asian international students were from Asian countries, non-Asian international students were from all other counties.	Self-injury and suicidal thoughts/behaviors were assessed based on participants’ responses to three questions regarding the past 12 months: “Self-injury” (Yes/No) “Considered suicide” (Yes/No) “Attempted suicide”(Yes/No)	Asian international students reported higher rates of self-injury (5.3%), suicidal ideation (5.6%), and suicidal attempts (2.1%) than American domestic students (3.2%, 5.2%, and 0.8%) and non-Asian international students (4.3%, 5.2%, and 1.1%), respectively. Whether these differences were statistically significant was not specified.	Not reported
41, 42 (two studies with the same sample) The United States CAQQS: 81.8% & 81.8%	To test a stress-problem-solving model and a stress-social support model in the etiology of depressive symptoms, hopelessness, and suicide ideation for a group of Asian international students in the United States.	101 Asian international students (Age M = 23.49, SD = 4.48, 27.72% female, 72.3% male).	India (29), China (21), Indonesia (10), Vietnam (9), South Korea (6), Taiwan (4), Malaysia (3), Philippines (3), Pakistan (3), Hong Kong (2), Thailand (2), Iran (2), Japan (1), Singapore (1), Bangladesh (1), Sri Lanka (1), Israel (1), Lebanon (1), Turkey (1).	Suicidal ideation - Modified Scale for Suicide Ideation (MSSI), which contains 18 items on a 4-point Likert-Scale from 0-3 that utilize specific anchors for each question, range = 0-54. Measured in the past 2 weeks.	21.8% reported suicidal ideation (score of 4 or more on the MSSI): M = 2.50. SD = 6.07.	Low social support, depressive symptoms, low problem-solving confidence, hopelessness, and life stress were positively associated with suicidal ideation. Problem-solving confidence indirectly influenced suicidal ideation via depressive symptoms and hopelessness. Social support moderated the relationship between life stress and suicidal ideation; those experiencing more life stress with low social support were likely to have higher suicidal ideation.
43 The United States CAQQS: 81.8%	To assess the prevalence and correlates of mental health symptoms and diagnoses in domestic and international college students studying in the United States.	44,851 undergraduate students. Age statistics not reported. International = 2423 (37.8% male, 59.3% female, 2.5% other). Domestic = 42,428 (29.3% male, 67.8% female, 2.5% other).	Race was reported instead of origin country for the international group. The break is as follows: 26.5% White, 7.9% Hispanic, 6.2% Black, 44.8% Asian, 0.1% American Indian/Alaskan Native/Native Hawaiian, 10.0% Multiracial.	Study-specific measure where participants reported behavior frequency in the past 12 months: Self-harm - “Intentionally cut, burned, or otherwise injured yourself” (Yes/No) Suicidal ideation - “Seriously considered suicide” (Yes/No) Suicide attempt - “Attempted suicide” (Yes/No)	12-month prevalence rates in international students were 7.7% for self-harm, 9.8% for suicidal ideation and 2.2% for suicide attempts. International students reported significantly lower rates of suicidal ideation, NS different rates of self-injurious behavior, and significantly higher rates of suicide attempt, compared to domestic students.	Not reported
44 The United States CAQQS: 77.3%	To better understand common mental health concerns and service utilization among international students.	228,421 university students (9.80% international). Domestic: 68.63% aged 18-22, 12.28% aged 23-25, 9.87% aged 26-30, and 9.22% aged 31+. 65.03% female. International: 40.43% aged 18-22, 25.12% aged 23-25, 23.24% aged 26-30, 11.22% aged 31+. 54.88% female, 44.22% male, and.90% non-binary.	China (n = 5,458), India (n = 2,432), South Korea (n = 903), Canada (n = 619), Brazil (n = 361), Saudi Arabia (n = 346), and Spain (n = 327). Total 151 countries with remainder containing less than 300 students per group.	Suicidal ideation was assessed by one item that asked participants if they have seriously considered suicide in the past year. NSSI - measured by asking participants to identify “ways you may have hurt yourself on purpose, without intending to kill yourself” in the past year from a list of eleven means (e.g., cut myself). Responses were dichotomized to indicate whether there was any NSSI or not in the prior 12 months.	8.8% of international students reported suicidal ideation (China = 8.2%, India = 8.7%, South Korea = 12.6%, Canada =7.6%, Brazil = 7.4%, Saudi Arabia 9.2%, and Spain = 7.0%). 17.2% of international students reported NSSI. (China = 15.8%, India = 16.5%, South Korea = 21.2%, Canada =16.4%, Brazil = 16.7%, Saudi Arabia = 15.5%, and Spain = 15.1%). International students had lower rates of suicidal ideation or NSSI compared to domestic students.	Female international students endorsed a significantly higher rate of suicidal ideation (9.9% vs. 7.0%) and NSSI (17.6% vs. 16.1%), compared to males. South Korean international students had the highest rates of suicidal ideation and NSSI out of all student groups, however, this was not tested for statistical significance.

CAQQS, Checklist for Assessing the Quality of Quantitative Studies (19); NS, Non-significant; PHQ-9, Patient Health Questionnaire-9 (45); NSSI, Non-suicidal self-injury; C-SSRS, Columbia Suicide Rating Scale (46); BSSI, Beck Scale for Suicide Ideation (47); SIS, Suicidal Ideation Scale (48).

1, This score was not reported in the paper, but calculated from the associated dataset that was included with the publication; 2, Mean is 1.30 for the normalized scale, so on the original scale (range 10-50) it would have been 13.00.

### Characteristics of included studies

Of 24 studies, 21 were published between 2013 and 2023, and three prior to that (34, 41, 42). Fifteen studies were conducted in the USA; the remainder came from Asia (3), Australia (3), Europe (2), and Canada (1). There were 21 cross-sectional studies, one case series (24), one longitudinal (26), and one case-control study (40). The included studies scored between 63.6% and 95.5% on the CAQQS tool. Most studies were not guided by a suicide-specific theoretical framework, while four (32, 33, 36, 39) referenced the Interpersonal Theory of Suicide (49).

Ten studies consisted solely of international students, and fourteen had an international student sub-sample and reported their demographic information separately. Because Yang and Clum’s two studies (41, 42) used the same sample, from hereon it will be counted only once when reporting demographic data and suicide-related outcomes. The sizes of international student samples and sub-samples ranged from 46 to 22,385 with a median of 334.0. Where international students comprised a sub-sample, their proportion of the overall sample ranged from 4.6% to 75.0% ( $\bar{x}$ = 30.1%).

On average, international student samples had a slightly higher proportion of females (range = 27.7-68.0%, $\bar{x}$ = 56.1%) than males (32.0-72.3%, $\bar{x}$ = 43.9%). Twelve studies reported mean ages of international students (range = 20.2-28.5 years, $\bar{x}$ = 23.7). Six studies reported only age ranges (18-30+), and five did not report age. All samples consisted of college or university students at the undergraduate or postgraduate level. Most international students were from Asia, with China and India being the most common countries of origin.

### Prevalence and intensity of suicidal thoughts and behaviors

#### Suicidal ideation

Nineteen studies reported on suicidal ideation in international students using a diverse range of measures and timeframes. In terms of prevalence rates, one study found that 46.0% of students presenting at a psychiatric emergency clinic reported suicidal ideation in the past week, measured by the Columbia Suicide Severity Rating Scale (C-SSRS) (24). The proportions of students endorsing suicidal ideation in the past two weeks were 28.6% (22) and 23.4% (32) when measured by item 9 from the Patient Health Questionnaire-9, and 21.8% when measured by the Modified Scale for Suicide Ideation (41, 42). According to one study, 6-month prevalence of suicidal ideation was 11.2%, measured by the C-SSRS (26). Five studies reported on prevalence in the past year, yielding a range of 5.4-18.2% (23, 37, 40, 43, 44). This was measured by various purpose-built questions or items borrowed from existing measures. Prevalence of suicidal ideation since commencing study abroad was 11.3% in females and 6.7% in males, as measured by a purpose-built question (34). Lifetime prevalence of suicidal ideation was 26.0% (26).

Five studies measured the intensity of suicidal ideation in the past week. The mean scores were 13.00 and 12.99 on the Suicidal Ideation Scale (full range 10-50) (33, 39), 3.11 on the Beck Scale for Suicide Ideation (BSSI; full range 0-42) (27), 0.38 on the first five items of the BSSI (full range 0-10) (29), and 1.14 on a purpose-built question (full range 1-5) (21). The mean score for Jun and colleagues’ (25) sample on the BSSI was 11.30, but no timeframe was reported.

Five studies compared prevalence rates of suicidal ideation between international and domestic students. International students had significantly lower rates of suicidal ideation than domestic students in three of those studies (23, 43, 44) and similar rates to domestic students in the two remaining studies (24, 26). There was an additional study where international students reported higher rates than domestic students, but it was not stated whether this difference was statistically significant (40).

Three studies compared the intensity of suicidal ideation between international and domestic students. International students had similar intensity levels to domestic students in two studies (21, 36) and significantly higher levels than domestic students in one study (27).

#### Suicide attempts

Six studies provided data on prevalence rates of suicide attempts in international students. In one study, the rate of suicide attempts in the past week was 12.7% (24). In another study, 6-month prevalence was 3.5% (26). Four studies reported rates of attempts in the past year, ranging from 1.2% to 2.2% (23, 37, 40, 43). As for lifetime suicide attempts, the prevalence was 7.5% (26), while in another study with students presenting to a psychiatric emergency clinic, lifetime prevalence of multiple attempts (2+) was 14.3% (24). Four studies assessed suicide attempts using purpose-built questions that were not part of existing measures, and the remaining two used the C-SSRS (24, 26).

Five studies compared international and domestic students on the prevalence of suicide attempts, yielding mixed results. Rates over the past year were significantly higher in international than domestic students in two studies (23, 43). There was a third study where international students also reported higher suicide attempt rates than domestic students, but this difference was not tested for significance (40). In one study, international and domestic students did not differ on lifetime prevalence of attempts at entry to college, but at 6-month follow-up international students were significantly more likely to have made an attempt than domestic students (26). Another study reported that while international and domestic students accessing psychiatric services did not differ on past-week attempt rates, international students had a higher lifetime prevalence of multiple attempts than domestic students (24).

#### Suicides

No studies reported rates of completed suicide.

#### Self-harm

Six studies measured and reported self-harm rates in international students. Among students presenting at a psychiatric emergency clinic 12.7% reported engaging in self-harm in the past week (24), whereas 6-month prevalence of self-harm was 5.6% in a general university sample (26). Three studies reported on past-year rates of self-harm, which ranged from 4.8% to 17.2% (40, 43, 44). Prevalence of self-harm since commencing study abroad was 4.5% in females and 2.0% in males (34). Lifetime prevalence was 13.0% in one study (26), while 25.4% of international students presenting to psychiatric services reported a lifetime history of multiple self-harm instances (2+) (24). Five of the six studies used purpose-built questions as opposed to a standardized measure; the study by King et al. (26) used the C-SSRS.

Studies that compared the rates of self-harm in international and domestic students reported mixed results. Three studies found no difference in self-harm rates (24, 26, 43), while in one study international students had lower rates of self-harm than domestic students (44).

### Narrative synthesis of risk and protective factors for suicidal thoughts and behaviors

#### Suicidal ideation

Several factors related to social isolation emerged as risk factors for suicidal ideation. Three studies found an association between low social support and suicidal ideation (32, 41, 42). High social support was a protective factor, but this association was weaker for international than domestic students (32). Another factor related to suicidal ideation was low campus belongingness in two studies (29, 36), while unmet interpersonal needs (37) and loneliness (29) were factors identified by single studies. Conversely, high family belongingness emerged as a potential risk factor, with international students reporting higher suicidal ideation at higher levels of family connectedness (36).

There were numerous intrapersonal factors relating to a person’s psychological functioning that emerged as risk factors. Three studies found a relationship between depression and suicidal ideation (25, 39, 41), while single studies identified the following as risk factors: generalized anxiety, social anxiety, eating concerns, hostility, maladaptive perfectionism (25); personal discrepancy between desired and actual educational performance (39); low problem-focused coping skills and hopelessness (41).

Several contextual factors related to an individual’s external setting emerged as risk factors. Suicidal ideation was associated with perceived discrimination in three studies (33, 37, 39), academic stress in two studies (25, 33), life stress in two studies (29, 41), and family discrepancy between desired and actual educational performance in two studies (25, 39). Single studies identified a relationship of suicidal ideation with the following factors: cross-cultural loss (33); perceived public stigma (23); residing with family, perceived susceptibility to COVID-19, and perceived insufficiency with resources needed to prevent COVID-19 (21).

There was mixed evidence for several potential risk factors. There were no gender differences on suicidal ideation in two studies (21, 36), but in Zhou and colleagues’ study (44) rates of suicidal ideation were significantly higher in females (9.9%) than males (7.0%). Similarly in King et al. (26), more females than males reported suicidal ideation at both entry to college (35.3% vs. 20.5%) and at 6-month follow-up (15.3% vs. 7.5%), but this difference was not tested for statistical significance. Of the three studies that assessed perceived burdensomeness, two reported a significant association (33, 39) and one did not (36). Additionally, several factors correlated with suicidal ideation but did not predict it when using predictive models (e.g., regression analyses or structural equation modelling), namely thwarted belongingness (33, 39), cultural stress, family conflict, feelings of entrapment, and perfectionism (37).

#### Suicide attempts

Two studies assessed risk or protective factors related to suicide attempts. Higher perceived public stigma was associated with greater incidence of attempts, and this association was stronger in Asian international than white students (23). Among international students, males had a higher prevalence of attempts than females, but whether this difference was significant was not reported (26). Being a Chinese versus non-Chinese international student was not associated with differing prevalence of attempts (26).

In a study that assessed suicidal ideation and attempts as a combined variable, being non-heterosexual significantly predicted suicidality (30).

#### Suicides

No risk or protective factors for completed suicides were reported.

#### Self-harm

Two studies assessed risk and protective factors for self-harm behavior, while others focused on self-harm thoughts, or a combination of self-harm thoughts and behaviors. Female international students reported higher self-harm rates than males in two studies; 17.6% vs. 16.1% (statistically significant) (44) and 4.5% vs. 2.0% (not tested for significance) (34). Furthermore, significantly more female than male students endorsed an item asking if they have engaged in and/or thought about self-harm (28). One study found that international students who engaged in self-harm had significantly higher scores on depression, anxiety, stress, and experiences of abuse (and associated distress) than those who had not self-harmed (34). Loneliness and perceived discrimination were significantly associated with greater odds of self-harm thoughts, while the following covariates were not associated: gender, age, relationship status, mask-wearing, and being Asian (38).

## Discussion

This review examined the prevalence of, and risk and protective factors for, suicidal thoughts and behaviors in international students. A total of 24 studies were included with the majority published since 2013. Nineteen studies reported data on suicidal ideation, six reported on suicide attempts, and seven addressed self-harm. No studies reported on suicide deaths. In terms of quality appraisal, the studies scored moderate to high on the CAQQS, suggesting that the data quality is acceptable. However, the current review highlights shortcomings in research on suicide-related thoughts and behaviors among international students, including a lack of studies and diverse methodologies leading to inconsistent findings.

### Key findings

The prevalence rates of suicidal ideation, attempts and self-harm varied depending on the instruments used, the measurement timeframe, and the nature of the sample. With the exception of international students presenting at a psychiatry clinic (24), rates of suicidal ideation (5.4-28.6%), suicide attempts (1.2%-7.5%), and self-harm (4.8%-17.2%) across timeframes, populations and measures were similar to rates from general college populations (50). Suicidal ideation intensity was typically low among international students, but the inclusion of cases without suicidal ideation may have positively skewed results.

Specific comparisons between international and domestic students produced mixed results. International students reported lower rates, but similar intensity of suicidal ideation compared to domestic students. In contrast, international students displayed a higher prevalence of suicide attempts than their domestic counterparts. Self-harm rates were similar in domestic and international students.

Similar to prior research on common stressors for international students (7), factors related to social isolation emerged as a common risk factor for suicidal ideation. This aligns with the Interpersonal Theory of Suicide (49), which proposes that thwarted belongingness, encompassing various elements related to social connectedness and the need to belong, is strongly related to suicidal thoughts and behaviors. Surprisingly, family connectedness was positively linked to increased suicidal ideation, contrasting with other social connectedness variables. This might be due to the distance from family networks during international studies, suggesting that factors related to social connectedness and isolation are not a homogenous group. More research is required to determine which factors relate to suicidal ideation and the time of their impact, with longitudinal studies needed to establish causality in these relationships.

Other significant risk factors for suicidal ideation encompass comorbid mental health conditions like depression and anxiety, contextual stressors such as experiences of racism and academic stress, along with inadequate coping abilities. These risk factors bear similarities to those observed in other student populations and provide potential opportunities for targeted intervention strategies. An emerging risk factor from the recent years was COVID-19 related stressors (21). International students are a population whose mental health was particularly impacted by COVID-19 restrictions (51), and the present review shows that COVID-19 related stressors are also specifically linked to suicidal thoughts and behaviors. As such, suicide prevention strategies for international students need to incorporate these common risk factors, including potential past and ongoing impacts of the COVID-19 or other pandemics.

The only significant risk factor for suicide attempts was high perceived public stigma. Given that suicidal ideation and attempts are closely related concepts, it may be that risk and protective factors for ideation are also applicable to attempts. However, more research is needed to confirm this. Risk factors for self-harm included identifying as female and common mental health symptoms like depression and anxiety. However, each of these were tested within individual studies so further evidence substantiating these associations is needed. Future studies should also differentiate self-harming behavior from thoughts of self-harm.

### Measurement issues in the literature

Various measurement tools and timeframes were used for suicidal ideation, attempts, and self-harm, as well as risk and protective factors. This diversity introduced complexity in synthesizing and comparing research findings. Some studies also measured certain outcomes but did not report them. Furthermore, in several studies (28, 30, 35), the outcome measures were worded in a way that made it impossible to distinguish between different suicide or self-harm related outcomes, for example, using questions like “Have you considered or attempted suicide?” (Yes/No). As such, these studies could not be included in our synthesis of prevalence rates or risk and protective factors. Future studies should use validated assessment tools that clearly differentiate between suicidal ideation, suicide attempts, and self-harming behaviors (52).

When reporting the intensity of suicidal ideation, studies presented average scores on various instruments. These average scores were often low due to most participants in the sample reporting no ideation. Because the average score is skewed by the large number of zero scores, it is impossible to discern if the participants who scored above zero reported low or high levels of ideation. Future studies should consider reporting suicidal ideation data for only those who scored above zero to better capture intensity for those experiencing suicidal thoughts. Addressing these limitations would facilitate an improved understanding of suicidal thoughts and behaviors in international student cohorts.

### Implications and future directions

This review showed consistent evidence for social connectedness as a protective factor against suicidal ideation. Promoting social connectedness is already part of university-wide suicide prevention frameworks (53). However, tailored approaches may be required for international students given that they are separated from their social support networks from their home country and may struggle to make new connections due to language barriers or cultural differences (54). Students’ sense of connectedness can be improved by socializing with co-nationals and other international students (54), and as such, universities should continue investing in social activities designed to help international students connect with peers.

Similarly, our review identified common mental health concerns like depression and anxiety as risk factors for suicidal thoughts and behaviors. Access to mental health care is imperative in reducing these symptoms and subsequently lowering the risk of suicide (55). However, it is well-documented that international students underutilize mental health services despite needing them (11). Evidence suggests there are multiple barriers to service access for international students, including low mental health literacy, a lack of culturally appropriate or accessible services, and high costs (13). These are all areas for improved prevention. For instance, international students are often unaware that mental health support is covered by their health insurance, which may be a barrier to help-seeking due to the assumed cost of services (56). International student offices and insurers should ensure that students stay adequately informed about the extent of their mental healthcare cover. Furthermore, mental healthcare pathways for international students should be streamlined across universities, community psychological services and emergency departments (12).

None of the studies included in this review reported on suicide deaths in international students. It is recommended that educational institutions adopt a specific reporting framework for international student suicides (57). Quantifying the rates of suicide mortality is imperative for tracking the scope of the issue on local and global scales, and guiding prevention efforts, as they can provide direct insight into potential causal factors in in such deaths (11, 58).

Additionally, the literature rarely reported the length of time international students have spent in the host country. There is evidence that being new to the country is associated with elevated suicide risk; a coroner’s investigation into international student suicides in Victoria, Australia found that almost half of individuals that took their lives had been in the host country for less than two years (12). Therefore, future studies should investigate the effect of time spent in the host country on suicide-related outcomes.

Future research should explore suicide-related outcomes among diverse international student subgroups to enhance the generalizability of findings, as the current studies have limited representation of countries and ethnicities. International students are a heterogeneous population, and such comparisons are essential. There is preliminary evidence suggesting potential subgroup differences, particularly that Asian international students report higher rates of suicidal ideation, attempts, and self-harm than other international students (40, 44).

There is currently no evidence on effective suicide prevention initiatives for the international student populations (13). However, based on the findings on risk and protective factors from this review, we suggest that emphasis should be placed on promoting social connectedness and improving student mental health via better access to psychology services. Combatting stigma and discrimination that international students experience and managing academic and general life stress are also areas for prevention. Generally, suicide prevention efforts will be most effective when undertaken collaboratively across educational institutions, mental health services, government, and other organizations relevant to international students (13). It is also essential to involve international students and those who work closely with them in designing and implementing suicide prevention initiatives (56).

### Strengths and limitations

To authors’ knowledge, this is the first publication to summarize the rates of suicidal ideation, attempts and self-harm in international students across the existing body of literature. Furthermore, this review describes prominent risk and protective factors for suicidal thoughts and behaviors in international students and highlights gaps for future research in this area.

The main limitation of this review is that currently there is not enough available data to draw any major conclusions regarding prevalence rates or factors contributing to suicidality in this student group. It was also not possible to conduct a meta-analysis to quantify the available data due to the diverse range of measures in the primary studies. In line with this, the available literature had varying measures of suicidal thoughts and behaviors, and varying overall methodology, meaning that the quality of data may vary between studies. Finally, it is possible that some studies relevant to this review were not captured by our search strategy.

## Conclusion

This review describes the prevalence rates, and risk and protective factors, for suicidal ideation, attempts, and self-harm in international students. Overall, there is little literature on this topic, limiting our ability to draw conclusions. However, in general, the literature suggests that international students may experience similar rates of suicidal ideation and self-harm, and higher rates of attempts compared to domestic students. Key risk factors include social isolation, intrapersonal factors (e.g., depression, anxiety), and contextual factors (e.g., perceived discrimination). More high-quality research featuring robust measurement is needed to adequately capture the prevalence of suicide-related outcomes in international students. Future research can also focus on subgroup comparisons within international students, and comparisons with domestic students. Risk and protective factors should be investigated further, particularly via longitudinal designs to establish causal relationships. Finally, this review provides directions for suicide prevention initiatives for international students, focusing on promoting social connectedness, improving student mental health, and investing in accessible and culturally sensitive mental health care. Given that international students comprise a significant portion of tertiary students worldwide and experience substantial levels of suicidal thoughts and behaviors, it is important that they receive targeted, appropriate, and effective support.

## Data availability statement

The original contributions presented in the study are included in the article/**Supplementary Material**. Further inquiries can be directed to the corresponding author.

## Author contributions

MV: Formal analysis, Investigation, Writing – original draft, Writing – review & editing, Data curation. ML: Project administration, Resources, Writing – review & editing. JR: Conceptualization, Funding acquisition, Writing – review & editing. SM: Conceptualization, Data curation, Funding acquisition, Investigation, Methodology, Project administration, Writing – review & editing.
