# Supplementary material for: A systematic review and narrative synthesis of prevalence rates, risk and protective factors for suicidal behavior in international students
Source: Front Psychiatry. 2024 Mar 14;15:1358041. doi: 10.3389/fpsyt.2024.1358041 (PMC10973160; doi:10.3389/fpsyt.2024.1358041)
Supplement: Supplementary file 1 [file DataSheet_1.pdf]

OVID BASED DATABASE SEARCH STRATEGY

Included databases to be searched via OVID:

- *MEDLINE(R) and Epub Ahead of Print, In-Process, In-Data-Review & Other Non-Indexed Citations and Daily*
- *APA PsycInfo*
- *Embase Classic+Embase*

Search strategy:

1. (Suicid* or Self-harm* or self-injur* or self-mutilat*).mp. [mp=title, abstract, original title, name of substance word, subject heading word, floating sub-heading word, keyword heading word, organism supplementary concept word, protocol supplementary concept word, rare disease supplementary concept word, unique identifier, synonyms]
2. (International student* or overseas student* or foreign student*).mp. [mp=title, abstract, original title, name of substance word, subject heading word, floating sub-heading word, keyword heading word, organism supplementary concept word, protocol supplementary concept word, rare disease supplementary concept word, unique identifier, synonyms]
3. (risk facto* or mechan* or predict* or protective facto* or facto* or influence or correlate* or precurs* or causal facto*).mp. [mp=title, abstract, original title, name of substance word, subject heading word, floating sub-heading word, keyword heading word, organism supplementary concept word, protocol supplementary concept word, rare disease supplementary concept word, unique identifier, synonyms]
4. (Epidemiology or incidence or prevalence or cohort study).mp. [mp=title, abstract, original title, name of substance word, subject heading word, floating sub-heading word, keyword heading word, organism supplementary concept word, protocol supplementary concept word, rare disease supplementary concept word, unique identifier, synonyms]
5. 3 OR 4
6. 1 AND 2
7. 5 AND 6

EBSCOHost BASED DATABASE SEARCH STRATEGY

Included databases to be searched via EBSCOHost:

- *Cumulative Index to Nursing and Allied Health Literature (CINAHL)*
- *Education Resources Information Center (ERIC)*

Search strategy:

1. Suicid* or Self-harm* or self-injur* or self-mutilat*
2. International student* or overseas student* or foreign student*
3. risk facto* or mechan* or predict* or protective facto* or facto* or influence or correlate* or precurs* or causal facto*
4. Epidemiology or incidence or prevalence or cohort study
5. 3 OR 4
6. 1 AND 2
7. 5 AND 6
