# Supplementary material for: A systematic review and narrative synthesis of prevalence rates, risk and protective factors for suicidal behavior in international students
Source: Front Psychiatry. 2024 Mar 14;15:1358041. doi: 10.3389/fpsyt.2024.1358041 (PMC10973160; doi:10.3389/fpsyt.2024.1358041)
Supplement: Supplementary file 2 [file DataSheet_2.docx]

**Supplementary file B: Data extraction template**

**General information**

**Country in which Study was conducted:**

- United States
- UK
- Canada
- Australia
- Other:

*Notes:* Add any additional information here that you think may be helpful and is not included in any other sections

|  |
| --- |

**Characteristics of included studies**

**Aim of study:** *Copy/summarise aim of study*

|  |
| --- |

**Study design:**

- Randomised controlled trial
- Non-randomised experimental study
- Cohort study
- Cross sectional study
- Case control study
- Systematic review
- Qualitative research
- Prevalence study
- Case series
- Case report
- Diagnostic test accuracy study
- Clinical prediction rule
- Economic evaluation
- Text and opinion
- Other:

**Start date:** *List when the study started - if unclear note NR (not reported)*

|  |
| --- |

**End date:** *List when the study ended - if unclear note NR (not reported)*

|  |
| --- |

**Study funding sources:**

|  |
| --- |

**Possible conflicts of interest for study authors:**

|  |
| --- |

**Participants**

**Population description:** *Describe the type of population/s e.g., University students. If multiple groups number each group e.g., Group 1 = etc.*

|  |
| --- |

**Inclusion criteria:** *Describe any inclusion criteria for participants in the study*

|  |
| --- |

**Exclusion criteria:** *Describe any exclusion criteria for participants in the study*

|  |
| --- |

**Method of recruitment of participants:**

- University international student department
- University health clinic
- Email
- Online (e.g., social media/university meddage)
- Mail
- Phone
- Other:

**Total number of participants:** *Enter number of participants. If more than one group, list group then number e.g. Group 1 = 100 etc*

|  |
| --- |

**Age:** *Report age mean and SD for each group. If reporting multiple cohort groups, please make sure group numbers are consistent across responses*

|  | Mean | SD |
| --- | --- | --- |
| Group 1 |  |  |
| Group 2 |  |  |
| Group 3 |  |  |
| Group 4 |  |  |
| Group 5 |  |  |

**Gender:** *Report percentage of each gender group. If reporting multiple cohort groups, please make sure group numbers are consistent across responses*

|  | Male | Female | Other |
| --- | --- | --- | --- |
| Group 1 |  |  |  |
| Group 2 |  |  |  |
| Group 3 |  |  |  |
| Group 4 |  |  |  |
| Group 5 |  |  |  |

**Country of origin:** *Enter the home country of the participants*

|  | Country 1 | Country 2 | Country 3 | Country 4 | Country 5 |
| --- | --- | --- | --- | --- | --- |
| Group 1 |  |  |  |  |  |
| Group 2 |  |  |  |  |  |
| Group 3 |  |  |  |  |  |
| Group 4 |  |  |  |  |  |
| Group 5 |  |  |  |  |  |

**Country of origin notes:** *If more than 4 countries of origin per group in the study, please list them here*

|  |
| --- |

**Time in host country:** *Describe the amount of time international students had spent in the host country e.g., University students. If multiple groups number each group e.g., Group 1 = etc. If not reported, then note NR*

|  |
| --- |

**Study level:** *Describe the level of study participants were completing, e.g. undergraduate, postgraduate etc. If multiple groups number each group e.g. Group 1 = etc.*

|  |
| --- |

**Measures**

**List each measure/outcome:** *If no measures used, list NA for not applicable. If multiple time points, please list the measures at each time point e.g.: Pre-test: Suicidal ideation, Post-test: Suicidal ideation, Post-training distress*

|  |
| --- |

**Outcomes**

**Incidence/Prevalence rates of attempt/ideation/self-harm:** *Note any included incidence or prevalence rate information in the study - NR for not reported*

|  |
| --- |

**Risk factors:** *List any identified risk factors including key variables/concepts - NR for not reported*

|  |
| --- |

**Protective factors:** *List any identified protective factors including key variables/concepts - NR for not reported*

|  |
| --- |

**Research limitations and gaps:** *List any identified limitations or research gaps reported by study authors*

|  |
| --- |

**Recommendations including future research:** *What were the main take home messages for research, policy or practice*

|  |
| --- |
